# Predicting the risk of bone metastases in patients with newly diagnosed prostate cancer – Development and validation of a nomogram model

**DOI:** 10.1371/journal.pone.0318051

**Published:** 2025-01-30

**Authors:** Wei Xu, Guoyu Zhu, Xiaoxiang Wang, Xuebing Yan, Fujun Wang, Shanyi Li, Wenji Li

**Affiliations:** 1 Danyang Hospital of Traditional Chinese Medicine, Zhenjiang, Jiangsu, PR. China; 2 Institute of Translational Medicine, Medical College, Yangzhou University, Yangzhou, Jiangsu, PR. China; 3 Jiangsu Key laboratory of integrated traditional Chinese and Western Medicine for prevention and treatment of Senile Diseases, Yangzhou University, Yangzhou, Jiangsu, PR. China; 4 Sino-Malaysia Molecular Oncology and Traditional Chinese Medicine Delivery Joint Research Centre, Medical College, Yangzhou University, Yangzhou, Jiangsu, PR. China; 5 Department of Urinary Surgery, The Affiliated Hospital of Yangzhou University, Yangzhou University, Yangzhou, Jiangsu, PR. China; 6 Department of Oncology, The Affiliated Hospital of Yangzhou University, Yangzhou University, Yangzhou, Jiangsu, PR. China; Memorial Sloan Kettering Cancer Center, UNITED STATES OF AMERICA

## Abstract

**Objectives:**

The aim of this study was to develop and validate a nomogram model that predicts the risk of bone metastasis (BM) in a prostate cancer (PCa) population.

**Methods:**

We retrospectively collected and analyzed the clinical data of patients with pathologic diagnosis of PCa from January 1, 2013 to December 31, 2022 in two hospitals in Yangzhou, China. Patients from the Affiliated Hospital of Yangzhou University were divided into a training set and patients from the Affiliated Clinical College of Traditional Chinese Medicine of Yangzhou University were divided into a validation set. Chi-square test, independent sample t-test, and logistic regression were used to screen key risk factors. Receiver operating characteristic (ROC) curves, c-index, calibration curves, and decision curves analysis (DCA) were used for the validation, calibration, clinical benefit assessment, and external validation of nomogram models.

**Results:**

A total of 204 cases were collected from the Affiliated Hospital of Yangzhou University, including 64 cases diagnosed as PCa BM and 50 cases collected from the Affiliated Clinical College of Traditional Chinese Medicine of Yangzhou University, including 12 cases diagnosed as PCa BM. Results showed that history of alcohol consumption, prostate stiffness on Digital rectal examination(DRE), prostate nodules on DRE, FIB, ALP, cTx, and Gleason score were high-risk factors for BM in PCa and nomogram was established. The c-index of the final model was 0.937 (95% CI: 0.899–0.975). And the model was validated by external validation set (c-index: 0.929). The ROC curves and calibration curves showed that the nomogram had good predictive accuracy, and DCA showed that the nomogram had good clinical applicability.

**Conclusions:**

Our study identified seven high-risk factors for BM in PCa and these factors would provide a theoretical basis for early clinical prevention of PCa BM.

## 1. Introduction

According to GLOBOCAN 2020, prostate cancer (PCa) is the second most common cancer in men worldwide and the fifth leading cause of cancer death in men [1]. In 2020, about 1414,259 cases of PCa were newly diagnosed worldwide, including 375,304 cancer-related deaths [1]. Current epidemiological surveys generally agree that PCa incidence is lower in Asian countries than in Western countries [2,3]. According to GLOBOCAN 2020, the age-standardized incidence rate of PCa in Chinese men in 2020 was 10.2/100,000, and the mortality rate of PCa patients in China was about 4.6/100,000, which were significantly lower than those in Western countries [1]. However, due to the large population in China, new cases of PCa in China account for 8.2% of the world and 13.6% of deaths [1], both indicators suggest that PCa is becoming a killer of men ‘s health in China.

It has been shown that PCa can be metastasized to different organs, but is more likely to metastasized to bone [4]. The spine, pelvis, and ribs are the most common sites of bone metastases [5]. Although PCa has a long course and slow progression, patients often have no specific discomfort at the beginning of the disease, the onset is occult, and the clinical progression of individual patients is uncertain, the tendency to develop bone metastases remains strong. Although the primary tumor can be detected early in many patients, bone metastases have been detected in up to 10% of patients with a primary diagnosis of PCa. In addition, 20% to 30% of patients with T1-T3 PCa relapse and develop advanced disease after radical prostatectomy, and bone metastases may occur in 70% to 80% of these patients [1]. Therefore, it is particularly important to predict the risk of developing bone metastases early and intervene early.

Previous studies has indicated that the risk factors for bone metastasis (BM) in PCa include age, tumor stage, chemoradiotherapy, Gleason score, PSA, etc. [6–9]. However, as they paid little attention to the patients’ personal life history and the clinical physical examination results, those findings encounters difficulties in clinical application. In this study, several factors affecting the occurrence of BM in PCa were obtained by using the data of clinical patients to predict the risk of BM in PCa early, providing a theoretical basis for the clinical prevention of BM treatment.

## 2. Patients and methods

### 2.1. Patients and data collection

We retrospectively collected data from two hospitals through the electronic medical record system on patients diagnosed with prostate cancer and prostate cancer bone metastasis between January 1, 2013 and December 31, 2022.Among them, the clinical data obtained from the Affiliated Hospital of Yangzhou University were training datasets for analyzing and constructing nomogram models, and the data from the Affiliated Clinical College of Traditional Chinese Medicine of Yangzhou University were validation datasets for model validation. The study was conducted in accordance with the Declaration of Helsinki. It was reviewed and approved by the Ethics Committee of Medical College at Yangzhou University (Ethics No.YXYLL-2021-148) and we are allowed to carry out this study from June 1, 2021 to June 30, 2022.

#### 2.1.1. Inclusion and exclusion criteria.

The inclusion and exclusion criteria of patients in the two groups followed the following criteria: 1. Inclusion criteria: PCa confirmed by prostate biopsy or surgery; Emission computed tomography (ECT) examination of systemic bone scan showed BM or no BM; Newly diagnosed cases; 2. Exclusion criteria: Patients with previous bone-related diseases; Patients with other malignant tumors; Patients with other malignant tumors and received radiotherapy, chemotherapy, immunotherapy and other treatments; Bone metastases caused by other malignant tumors; Patients who have experienced fracture, surgery and other related treatments within one month before the initial diagnosis of PCa.

Since patients with previous bone related diseases or tumor will affect the accuracy of hematological indicators and thus affect the construction of the final model, they were excluded from the study. The purpose of setting these exclusion criteria is to exclude those patients who have bone related diseases themselves, because patients have bone related diseases and PCa, and their bone related diseases will affect the accuracy of hematological indicators and thus affect the construction of the final model, so relevant criteria are set.

#### 2.1.2. Data collection.

Relevant data were exported and collected from the hospital information system(HIS). 1. Pathological data: The specimen sections of all patients were made in the data collection hospital, and were diagnosed as PCa by the pathologist of the hospital, and graded according to the Gleason grading criteria to obtain the Gleason Score; 2. Laboratory parameters: The blood routine, coagulation function, liver function, prostate tumor indicators of the patients before prostate puncture or before surgery were collected. All relevant indicators are the data detected by the clinical laboratory of the hospital where the data were collected. Other relevant modeling indicators were obtained by further calculation; 3. Imaging indicators: prostate color ultrasound, abdominal computed tomography(CT), prostate magnetic resonance imaging(MRI) and whole-body bone scan were collected from the patients. All relevant reports were issued by the Department of Radiology of the data collection hospital. Bone metastases were confirmed by reading by the Radiologist of this hospital. At the same time, the clinical T stage of the patients was determined according to the imaging findings; 4. Other indicators: patient age, past medical history, personal history, physician physical examination results of the patients and other data were obtained from the patient ‘medical history data.

We retrospectively collected the clinical data of patients with PCa who were initially pathologically diagnosed by prostate biopsy or surgery obtained during a 10-year period from January 1, 2013 to December 31, 2022 in two hospitals. A total of 204 cases were collected from the Affiliated Hospital of Yangzhou University, including 64 cases diagnosed as PCa BM and 50 cases collected from the Affiliated Clinical College of Traditional Chinese Medicine of Yangzhou University, including 12 cases diagnosed as PCa BM.

### 2.2. Statistical analysis

Data analysis was performed using SPSS software (v.25.0) and R software (R-4.0.3) for visualization and further validation of the data. Among them, the measurement data were compared between groups and expressed as mean ± standard deviation (x̅ ± s) using the independent sample t-test. The enumeration data were expressed as rate (%) using the chi-square test. Univariate and multivariate logistic regression analysis were used to screen the predictors, and Hosmer-Lemesho tested the goodness of fit of each logistic regression model. R software was used to construct nomogram models and subsequent validation. The C-index and calibration curve were used to evaluate the accuracy of the nomogram. In addition, we also plotted the ROC curve and the area under the curve (AUC) to evaluate the accuracy of the nomogram. DCA identifies and compares clinical value between nomogram model and other clinical features by calculating the net benefit at each risk threshold probability. *P-*value < 0.05 was considered statistically significant.

## 3. Results

### 3.1. Analysis between groups

As shown in Table 1, there was no statistically significant difference in age (p = 0.851), smoking history (p = 0.381), hypertension history (p = 0.078), diabetes history (p = 0.270), coronary heart disease history (p = 0.856), dysuria (p = 0.11), and prostate volume (p = 0.865) between groups. However, history of alcohol consumption (p = 0.028), stiffness of prostate on digital rectal examination(DRE) (p < 0.001), presence of nodules in prostate on DRE (p = 0.004), systemic inflammatory response index (SIRI) (p = 0.011), systemic immune-inflammation index (SII) (p = 0.004), fibrinogen (FIB) (p = 0.006), alkaline phosphatase (ALP) (p < 0.001), albumin alkaline phosphatase ratio (AAPR) (p = 0.001), total prostate specific antigen(tPSA) (p < 0.001), free prostate specific antigen(fPSA) (p < 0.001), prostate specific antigen density (PSAD) (p < 0.001), clinical t stage x (cTx) (p < 0.001), and Gleason Score (p = 0.042) were statistically significant between groups. Among them, the values of FIB, ALP, tPSA, fPSA, PSAD and Gleason Score in BM group were (3.60 ± 1.11) g/L(299.11 ± 388.02) U/L(233.18 ± 285.58) ng/ml(26.14 ± 22.91) ng/ml(5.29 ± 8.21) ng/ml^2^ and (7.92 ± 1.22) points, respectively, which were higher than those in non-BM group, while SIRI, SII and AAPR in BM group were lower than those in non-BM group.

**Table 1:** Between-group comparison of risk factors for PCa BM.

		Non-BM(n = 140)	BM(n = 64)	p value
Age, years		72.52 ± 8.41	73.78 ± 8.83	0.851
Smoking history	No	126 (61.8%)	60 (29.4%)	0.381
	Yes	14 (6.9%)	4 (2.0%)	
History of alcohol consumption	No	130 (63.7%)	53 (26.0%)	**0.028**
	Yes	10 (4.9%)	11 (5.4%)	
Hypertension history	No	58 (28.4%)	35 (17.2%)	0.078
	Yes	82 (40.2%)	29 (14.2%)	
Diabetes history	No	122 (59.8%)	52 (25.5%)	0.270
	Yes	18 (8.8%)	12 (5.9%)	
Coronary heart disease history	No	128 (62.7%)	59 (28.9%)	0.856
	Yes	12 (5.9%)	5 (2.5%)	
Dysuria	No	46 (22.5%)	14 (6.9%)	0.110
	Yes	94 (46.1%)	50 (24.5%)	
Prostate stiffness on DRE	Toughness	87 (42.6%)	14 (6.9%)	**< 0.001**
	Hard	53 (26.0%)	50 (24.5%)	
Prostate nodules on DRE	No	132 (64.7%)	52 (25.5%)	**0.004**
	Yes	8 (3.9%)	12 (5.9%)	
SIRI,/L		2.07 ± 3.68	1.23 ± 1.07	**0.011**
SII,/L		843.15 ± 1060.00	572.16 ± 336.46	**0.004**
FIB, g/L		3.06 ± 0.87	3.60 ± 1.11	**0.006**
ALP, U/L		89.57 ± 47.44	299.11 ± 388.02	**< 0.001**
AAPR, g/L		0.51 ± 0.16	0.44 ± 0.65	**0.001**
tPSA, ng/ml		54.72 ± 98.24	233.18 ± 285.58	**< 0.001**
fPSA, ng/ml		7.54 ± 9.94	26.14 ± 22.91	**< 0.001**
Prostate volume, ml		65.64 ± 52.89	72.96 ± 43.62	0.865
PSAD, ng/ml^2^		0.99 ± 1.85	5.29 ± 8.21	**< 0.001**
cTx	T2	115 (56.4%)	20(9.8%)	**< 0.001**
	T3	18 (8.8%)	29 (14.2%)	
	T4	7 (3.4%)	15 (7.4%)	
Gleason Score, points		6.93 ± 1.14	7.92 ± 1.22	**0.042**

The measurement data is expressed as mean ± standard deviation (x̅ ± s), and independent sample t-test is used. The counting data is expressed in rate (%), using chi-square test. The bold indicates that the parameter is p < 0.05, with statistical significance.

### 3.2. Logistic regression

We included factors with p < 0.05 in the between-group analysis in the logistic regression for further screening and the results are shown in Table 2 and 3. In Table 2, univariate logistic regression calculations showed a statistically significant regression of history of alcohol consumption (p = 0.033), prostate stiffness on DRE (p < 0.001), prostate nodules on DRE (p = 0.006), FIB (p < 0.001), ALP (p < 0.001), AAPR (p < 0.001), tPSA (p < 0.001), fPSA (p < 0.001), PSAD (p < 0.001), cTx (p < 0.001), and Gleason Score (p < 0.001) with PCa with or without bone metastases. Including the above indicators into multivariate logistic regression calculation (Table 3), it was found that history of alcohol consumption (p = 0.009), prostate stiffness on DRE (p =  0.025), prostate nodules on DRE (p = 0.002), FIB (p = 0.021), ALP (p = 0.007), cTx (pT3 = 0.006, pT4 = 0.034), and Gleason Score (p = 0.033) were risk factors for PCa BM.

**Table 2:** Parameters comparing between groups with p < 0.05 in logistic regression for univariate calculations.

		Univariate		
Parameters		β	p value	OR (95% CI)
history of alcohol consumption		0.993	**0.033**	2.698 (1.082–6.730)
prostate stiffness on DRE		1.769	**<0.001**	5.863 (2.958–11.618)
prostate nodules on DRE		1.337	**0.006**	3.808 (1.472–9.850)
SIRI,/L		−0.218	0.085	0.804 (0.628–1.030)
SII,/L		−0.001	0.053	0.999 (0.999–1.000)
FIB, g/L		0.559	**<0.001**	1.748 (1.279–2.390)
ALP, U/L		0.016	**<0.001**	1.016 (1.009–1.022)
AAPR, g/L		−3.697	**<0.001**	0.025 (0.005–0.130)
tPSA, ng/ml		0.007	**<0.001**	1.007 (1.004–1.009)
fPSA, ng/ml		0.079	**<0.001**	1.082 (1.055–1.110)
PSAD, ng/ml^2		0.337	**<0.001**	1.401 (1.193–1.646)
cTx	T2	ref		
	T3	2.226	**<0.001**	9.264 (4.350–19.727)
	T4	2.511	**<0.001**	12.321 (4.465–34.001)
Gleason Score, points		0.648	**<0.001**	1.912 (1.482–2.466)

Parameters obtained from comparisons between groups will be included in univariate logistic regression for calculation and those resulting in p < 0.05 will be included in multivariate logistic regression. Crude parameters marked in the table are those with p < 0.05.

**Table 3:** Parameters comparing between groups with p < 0.05 in logistic regression for multivariate calculations.

		Multivariate		
Parameters		β	p value	OR (95% CI)
history of alcohol consumption		1.941	**0.009**	6.963 (1.630–29.734)
prostate stiffness on DRE		1.091	**0.025**	2.976 (1.147–7.722)
prostate nodules on DRE		2.239	**0.002**	9.383 (2.348–37.498)
SIRI,/L				
SII,/L				
FIB, g/L		0.534	**0.021**	1.706 (1.085–2.680)
ALP, U/L		0.010	**0.007**	1.010 (1.003–1.017)
AAPR, g/L		−1.243	0.072	0.059 (0.013–1.332)
tPSA, ng/ml		0.016	0.134	1.114 (0.923–1.265)
fPSA, ng/ml		0.083	0.093	1.013 (0.855–1.127)
PSAD, ng/ml^2		0.583	0.084	1.251 (0.988–1.781)
cTx	T2			
	T3	1.609	**0.006**	4.996 (1.600–15.602)
	T4	1.639	**0.034**	5.150 (1.136–23.342)
Gleason Score, points		0.466	**0.033**	1.593 (1.039–2.442)

The final parameters were calculated by including the parameters obtained in univariate logistic regression in multivariate logistic regression. Crude parameters marked in the table are those with p <  0.05.

### 3.3. ROC curve and nomogram

**Fig 1:**
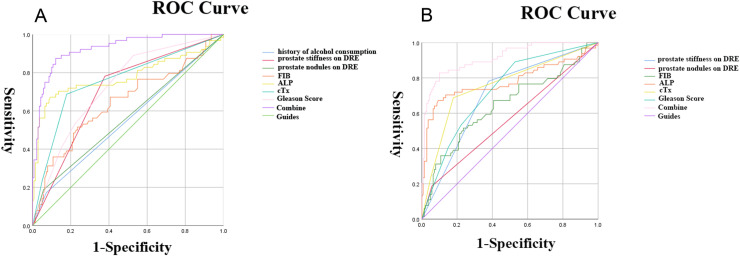
ROC curves constructed for parameters validated by logistic regression. (A). Seven parameters validated by multivariate logistic regression, the history of alcohol consumption, prostate stiffness on DRE, prostate nodules on DRE, FIB, ALP, cTx, and Gleason Score, were included in the calculation of ROC curves, and the AUC value of the final model was 0.927 (P < 0.05). (B). Subgroup analysis removing the factor drinking history revealed an AUC value of 0.914 for the ROC curve.

As shown in Fig 1A, the history of alcohol consumption, prostate stiffness on DRE, prostate nodules on DRE, FIB, ALP, cTx, and Gleason Score obtained in multivariate logistic regression were included in the construction of receiver operating characteristic(ROC) curves, and the predictive value of each factor for BM in newly diagnosed PCa patients was assessed according to the area under the curve. As shown in Table 4, the area under curve(AUC) values of the history of alcohol consumption, prostate stiffness on DRE, prostate nodules on DRE, FIB, ALP, cTx, and Gleason Score were 0.550, 0.701, 0.565, 0.645, 0.780, 0.758, and 0.729, respectively, which were lower than those when the seven were combined (AUC = 0.927), indicating that these seven played a synergistic role in the prediction of PCa BM and could be used as predictors of PCa BM together. Among them, we used the R package “ROCR” to calculate the Youden index, and the cutoff value corresponding to the maximum Youden index was the optimal diagnostic cut-off value. The Youden index of fibrinogen was 0.28 with a cutoff value of 3.495 g/L, and the Youden index of alkaline phosphatase was 0.579 with a cutoff value of 124.95 U/L. These two sets of cutoff values has been included in the model as stratification indicators for nomograms. As shown in Fig 1B, if history of alcohol consumption was excluded from the model, the AUC value of the combined model was 0.914, which was smaller than that of the combined seven.

**Table 4:** Table of AUC values for predictors of BM in PCa.

Parameters	AUC	95%CI
history of alcohol consumption	0.550	0.463-0.638
prostate stiffness on DRE	0.701	0.625-0.777
prostate nodules on DRE	0.565	0.477-0.653
FIB	0.645	0.559-0.731
ALP	0.780	0.698-0.862
cTx	0.758	0.682-0.834
Gleason Score	0.729	0.657-0.801
**Combine**	**0.927**	**0.888–0.965**
**Exclude history of alcohol consumption**	**0.914**	**0.87–-0.957**

The AUC values and their 95% CIs for the parameters included in the model are shown in the table.

**Fig 2:**
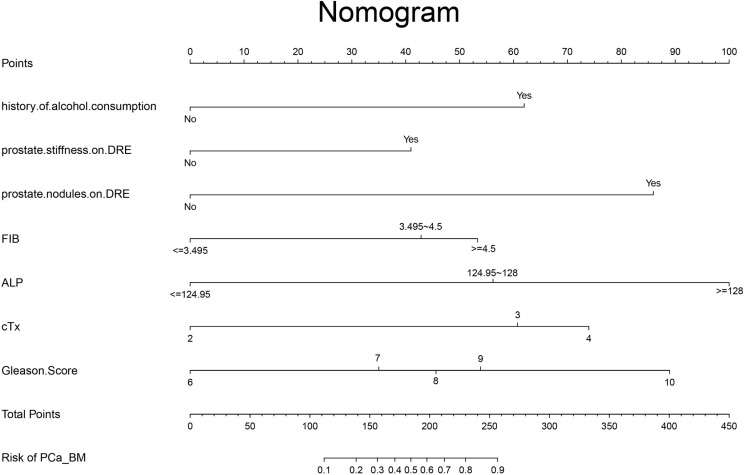
Nomogram constructed with parameters validated by ROC curve. Factors with p < 0.05 in multivariate logistic regression were included in the nomogram model. The c-index of the model was 0.937.

As shown in Fig 2, the history of alcohol consumption, prostate stiffness on DRE, prostate nodules on DRE, FIB, ALP, cTx, and Gleason Score obtained in multivariate logistic regression were included in the construction of the nomogram model.

### 3.4. Evaluation of nomogram

**Fig 3:**
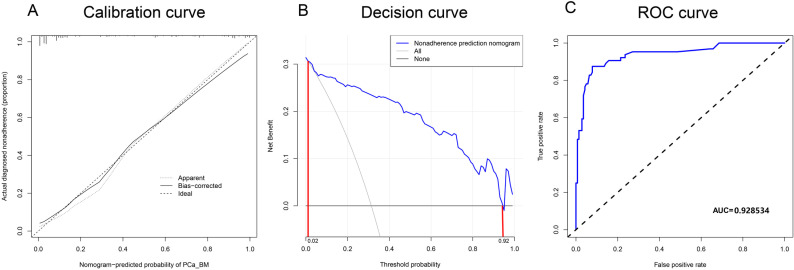
Re-validate nomogram model using calibration curve, decision curve, external validation set. (A). Calibration curve for nomogram model. The calibration curve of the model in this study is close to the ideal curve. (B). Decision curves for nomogram models. Intervention of patients within a threshold probability of 0.02 – 0.92 can be clinically beneficial. (C). ROC curves for the validation set validation nomogram model. The AUC value of the curve was 0.928534, indicating that the model predicted well.

As shown in Fig 3A, the calibration plot revealed good predictive accuracy between the actual probability and predicted probability. As shown in Fig 3C, the nomogram model used to predict PCa BM risk presented good agreement across the training set cohort. Meanwhile, the c-index of this nomogram model was 0.937 (95% CI: 0.899 – 0.975). The nomogram model also passed the validation of the validation set with a c-index of 0.929, which also illustrates the good predictive ability of the model.

### 3.5. Clinical benefit assessment

Fig 3B shows the decision curve for the nomogram model. The decision curve suggests that there may be more benefit in the clinical use of this nomogram model to predict prostate cancer bone metastasis risk if the threshold probability is between 0.02 and 0.92.

## 4. Discussion

BM is the most common site of metastasis in PCa, and early detection is important in clinical treatment. In our study, we used nomograms as modeling models, which are widely used in oncology and medicine to predict the risk or prognosis of diseases and can help better clinical decision-making. Seven clinically most common parameters, history of alcohol consumption, prostate stiffness on DRE, prostate nodules on DRE, FIB, ALP, cTx, and Gleason Score, were used in this study to participate in the construction of the nomogram model. These seven factors cover the personal history of patients, clinical examination results of doctors, blood biochemical results, coagulation function, imaging examination, pathological results, etc., which can comprehensively evaluate the physical status of PCa patients and their risk of BM, which is conducive to clinicians to apply and early predict and prevent the occurrence of BM of PCa. Bai G et.al. recently used six factors including total prostate-specific antigen, clinical tumor stage, Gleason score, prostate volume, red cell distribution width and serum alkaline phosphatase as the basis for constructing a nomogram model to predict the BM of PCa [9]. However, they did not include the analysis of the patient ‘s personal life history and the clinical physical examination results in their model, which will lose important information like the patient ‘s lifestyle habits on the disease and the clinical physical examination results, and hence sacrifice the accuracy of the prediction. In addition, we found that some articles discussed PCa and BM of PCa from the perspective of prostate MRI, which provided new ideas for the diagnosis and prognosis of prostate cancer and prostate cancer bone metastasis [10,11]. As far as the authors’ clinical practice is concerned, prostate magnetic resonance imaging is a very important test in urology, and in combination with changes in PSA levels, it is almost certain whether patients with benign prostatic hyperplasia or hypertrophy develop prostate cancer. It also plays an important role in cancer development and prognosis. Unfortunately, when reviewing the clinical data, it was found that many patients did not undergo MRI which is mainly due to the economic reason. Although MRI data were not evaluated, our model still passed the evaluation and validation of c-index, calibration curve and decision curve, demonstrating its good predictive ability. Our model also passed external validation, which demonstrates the validity of the model.

After univariate and multivariate logistic regression analysis (Table 2 and 3), it was found that uniquely, the history of alcohol consumption was included in the model as an important factor, which was less frequently identified as a key factor in previous studies. As shown in Fig 1B, if history of alcohol consumption was not included in the model, the AUC value of the model was lower than when it was included, indicating that history of alcohol consumption was an important factor in the model. Several studies have shown that long-term alcohol consumption can lead to oral cancer, pharyngeal cancer, laryngeal cancer, esophageal cancer, colorectal cancer, liver cancer, breast cancer, gastric cancer, pancreatic cancer, PCa and other cancers [12–15]. Alcohol, or acetaldehyde, can induce inflammatory responses and oxidative stress, and its metabolite acetaldehyde can lead to DNA damage as well as disrupt DNA methylation, all of which may lead to the development of cancer [16]. A study of the relationship between PCa and alcohol proposed that alcohol accelerates the growth of prostate tumors and significantly shortens the progression time of PCa metastasis [17], while alcohol reduces the effect of androgen deprivation therapy, resulting in the occurrence of PCa recurrence and metastasis [18–22]. The model included in this study may also be related to geographical reasons, and the proportion of men drinking alcohol has historically been higher in Jiangsu, so the probability of alcohol-induced diseases is also greater. Although the AUC value of history of alcohol consumption in this study was 0.550, which is insufficient to predict as an independent risk factor for BM in PCa, the effect on the model after inclusion in the model remains very large.

Hematological parameters also play an important role in PCa and PCa BM. In this study, FIB and ALP were found to be predictive factors for the diagnosis of bone metastases in PCa. FIB is highly expressed in various tumor tissues such as melanoma, lung cancer, renal cell carcinoma, and breast cancer, while it is most significantly expressed in breast cancer and is an independent risk factor for the prognosis of breast cancer patients [23]. FIB can provide structural support and scaffold for cancer cell metastasis and adhesion ligands for cell attachment and crawling [24], so FIB is equally important for the study of metastatic cancer. In the study of metastatic factors of gallbladder carcinoma, the experimenter found that FIB can promote tumor cell migration, cell adhesion, transendothelial cell migration, promote angiogenesis, increase vascular endothelial permeability, thereby promoting tumor cell metastasis and extravasation [25]. In a clinical analysis, a study of risk factors for bone metastases in patients with non-small cell lung cancer found that FIB existed as an independent risk factor [26]. In another study of the correlation between BM burden and FIB level in PCa, a positive relationship was found between FIB level and BM burden, and the higher the FIB level, the greater the probability of BM in PCa patients, and FIB was an independent risk factor for BM status [27]. Similarly, elevated expression levels of ALP are associated with the prognosis of a variety of malignancies [28–31] and ALP has prognostic value in castration-resistant PCa. High ALP levels have been shown to be associated with skeletal complications, lower survival rates, and bone metastases [32–34]. This is consistent with our findings that FIB and ALP can be included in the model as risk factors for BM in PCa. As drinking alcohol has been indicated to be related to the increase of alkaline phosphatase level [35–38], it justifies the reasonability of incuding both drinking history and alkaline phosphatase level in our nomogram model. Unfortunately, tPSA, fPSA, PSAD as indicators of PCa-specific tumor markers were not included in the model, which may be related to the small number of cases we collected, and we also need a large number of clinical samples as support for subsequent analysis. The same may exist, and important indicators commonly used to assess systemic inflammation, immunity, and nutritional status of patients, namely SIRI, SII, and AAPR, have not been included in the final model.

The cTx, Gleason Score, and DRE are extremely important factors for the diagnosis and treatment of PCa and judging the metastasis of PCa, as well as classical diagnostic factors. In our study, the three were also present as very important factors. It has been confirmed that the higher the T stage, the higher the Gleason Score, and the greater the risk of PCa BM [39], which is consistent with the results of this study. According to the nomogram model, the higher the T stage, the higher the Gleason Score, and the higher the score, the greater the risk of BM. ROC curves also showed the same characteristics. DRE has been the cornerstone of PCa detection [40]. Abnormal DRE is associated with an increased risk of PCa and is an important indication for PCa biopsy in both European and the United States guidelines for the diagnosis of PCa [41,42]. When PSA is < 3 ng/ml and there are abnormal DRE results, DRE can be used as a tool for multiple screenings [43–45]. At the same time, DRE results are one of the factors of a variety of classical clinical risk prediction calculation formulas, which include the European Randomised Study of Screening for Prostate Cancer risk calculator [46] and the Prostate Biopsy Collaborative Group calculator [47].

Overall, history of alcohol consumption may be included as the first time in PCa BM models, and it remains irreplaceable. Factors such as prostate stiffness on DRE, prostate nodules on DRE, FIB, ALP, cTx, and Gleason Score also play important roles in the construction of nomograms to predict the risk of prostate BM. Based on our nomogram model, especially through the novel key risk factors, the diagnostic accuracy of PCa BM can be improved greatly.

## 5. Limitations

Although our model predicts well, our study still has some limitations. First, limitations of the population. We selected PCa patients only from two of the three Class A tertiary hospitals in the local municipal area and did not include all similar patients from all hospitals in the area. Although these two tertiary hospitals are representative in this region, this model may not fully applicable to all PCa patients in China. Second, limitations of risk factors. We included PCa-related risk factors in detail, but parameters such as PSA could not be included in the final model due to population limitations, however this may also provide new ideas for the prediction of PCa BM. Third, limitations of applicability. Nomogram is only one of many prediction models. It has advantages such as easy to understand and apply clinically, however, it can still be improved in terms of prediction accuracy and multivariate processing ability. In our further studies, multiple machine learning, deep neural network, SHapley Additive ExPlanations and other methods are planed to be invloved to make improvements. Lastly, as this study is a retrospective study and we only selected the complete patient data. Missing data are therefore manageable. However, there still could exist unmeasured confounders. In the subsequent study, we will further adjust the selection strategy to strive for multi-regional, multi-center patient data in the hope of obtaining a better model.

## 6. Conclusion

Our study identified history of alcohol history, prostate stiffness on DRE, prostate nodules on DRE, FIB, ALP, cTx, and Gleason score as seven factors as high risk factors for PCa BM. Alcohol history was included in the model as a novel factor and played an important role in the model, and PCa patients with a previous history of alcohol consumption had a greater risk of BM. DRE of the prostate as a routine screening tool for PCa. Patients are at greater risk of later bone metastases if the prostate is firm to the touch or nodules are palpable on the prostate surface on DRE. Moreover, as shown in Fig 2, when FIB was greater than 3.495 g/L and ALP was greater than 124.95 U/L, FIB and ALP were present as high risk factors for PCa BM and were positively correlated with the degree of risk. At the same time, the more advanced the tumor stage and the higher the Gleason score, the greater the risk of secondary BM in PCa patients. The nomogram model involves four aspects: the basic situation of the patient, the physical examination of the clinician, the hematological examination results, and the imaging examination results. This allows a more comprehensive and accurate prediction of the likelihood of BM in PCa patients and is more conducive to clinical decision-making. In the future clinical application of this study model, it can be discussed in combination with prostate MRI results or biased to be applied in patients with a history of alcohol consumption, which can make the model more accurate.

## Formula table

**Table:** 

SIRI = monocyte count * neutrophil count/lymphocyte count
SII = neutrophil count * platelet count/lymphocyte count
AAPR = albumin/alkaline phosphatase ratio
PSAD = total prostate specific antigen/prostate volume

## Supporting information

S1 File Raw data. This file provided all of the information of patients diagnosed with prostate cancer and bone metastases of prostate cancer in this study.
(XLSX)

## Abbreviations:

AAPR: albumin alkaline phosphatase ratio
ALP: alkaline phosphatase
AUC: area under curve
BM: bone metastasis
CI: confidence interval
CT: computed tomography
cTx: clinical t stage x
DCA: decision curve analysis
DNA: deoxyribo nucleic acid
DRE: digital rectal examination
ECT: emission computed tomography
FIB: fibrinogen
HIS: hospital information system
fPSA: free prostate specific antigen
MRI: nuclear magnetic resonance imaging
PCa: prostate cancer
PSA: prostate specific antigen
PSAD: prostate specific antigen density
ROC: receiver operating characteristic
SII: systemic immune-inflammation index
SIRI: systemic inflammatory response index
tPSA: total prostate specific antigen.
